# Comparison of clinician- and patient-reported outcome measures in 95 abdominoplasty cases using BODY-Q and MCCRO-Q

**DOI:** 10.1016/j.jpra.2025.01.004

**Published:** 2025-01-10

**Authors:** Samuel Thomas Kitching, Claudia Rocco, Rachel Harwood, Gary Ross

**Affiliations:** 1 School of Medical Sciences, University of Manchester, Oxford Rd, Manchester, M14 4PX, UK; 2 Faculty of Social Sciences, University of Sheffield, Sheffield, S10 2SJ, UK; 3 Statistics Department, Research and Innovation, Manchester University NHS Foundation Trust, Manchester, M13 9PL, UK; 4 School of Biological Sciences, University of Manchester, Oxford Rd, Manchester, M13 9PL, UK

**Keywords:** Abdominoplasty, Patient-reported, Clinician-reported, Outcome measure

## Abstract

Patient-reported outcome measures (PROMs) and clinician-reported outcome measures (CROMs) are not routinely compared and they could be used to assess outcomes and aid patient selection and informed consent.

Between July 2016 and February 2020, we performed a cohort study comparing PROM versus CROM scores in 95 abdominoplasty cases with all patients undergoing psychological assessment using the Royal Free Hospital and Centre for Appearance Research (RoFCAR) screening tool.

Patients and clinicians described significantly improved outcomes from an abdominoplasty procedure (p<0.001; p<0.001), and patients also derived psychological benefits with improved RoFCAR scores (p<0.001). Patients reported a significantly greater improvement between pre- and post-operative outcomes as compared to the clinicians (p=0.017). Clinicians reported worse outcomes in patients with body mass index >30 kg/m^2^ or patients who had >1000 g of excess fat tissue removed (p=0.005; p=0.017). Clinicians reported better outcomes in patients who achieved massive weight loss through diet and exercise as opposed to bariatric surgery (p=0.044). Patients who underwent concomitant surgical operation had significantly improved clinician-scored outcomes (p=0.047), and patients with post-operative complications achieved worse clinician-scored outcomes (p=0.036). Pre-operative and post-operative scarring, previous pregnancy, and age >50 years did not affect clinician-scored outcomes. None of the tested factors significantly affected how the patients scored these outcomes.

We demonstrated that clinicians underestimate the improvement in outcomes described by the patients and they need to be aware of their selection bias when consulting with patients preoperatively, as patients reported improvement regardless of the pre-operative or post-operative variable tested.

## Introduction

Abdominoplasty is the third most common cosmetic procedure in the United States and the fourth most common in the United Kingdom, with cases increasing year on year (1,2). Comparison of patient- and clinician-reported outcomes can aid clinicians in managing patient expectations and their own expectations. Clinicians should be aware of the potential subgroups of patients who can view their results more favourably than clinicians and vice versa, which can aid in patient selection and consent acquisition. Standardised objective criteria to compare the results of an abdominoplasty operation will provide patients and clinicians with a standardised result that they can expect and compare to. Previous publications have reported outcomes via a satisfaction index based on the surgeon and patient reporting their overall impression on a scale of one (dissatisfied) to 5 (satisfied), with average satisfaction rates between 76% and 92% (3, 4, 5, 6). This approach failed to address the specific operation and did not provide sufficient information to explain the difference in scoring. Therefore, using operation or anatomically-specific questionnaires will increase the reliability of the responses (7).

The Royal College of Surgeons (RCS) recommends using the BODY-Q patient-reported outcome measures (PROMs) to assess abdominoplasty results, specifically the independent abdominal scale. It has demonstrated strong evidence for good content validity and moderate evidence for good internal consistency and structural validity (8, 9, 10). A recent systematic review of 24 PROMs found that BODY-Q had the strongest evidence for the quality of measurement in bariatric surgery and body contouring surgery patients (11).

The use of psychological screening in practice has been highlighted by the Care Quality Commission (CQC) and RCS. The senior author considers using psychological screening and PROM to be part of the standard practice. Using PROMs in practice has been highlighted by RCS and the Private Healthcare Information Network (PHIN) (12, 13, 14, 15).

There is no equivalent validated clinician-reported outcome measure (CROM) questionnaire to assess abdominoplasty procedures (16). The authors developed and validated the Manchester Cosmetic Clinical Reported Outcomes questionnaire (MCCRO-Q) (17). Previous comparisons of CROM and PROM scores showed a moderate correlation, and both provided clinical information on the functional state of the patient (18, 19, 20). However, a meaningful comparison between the BODY-Q and MCCRO-Q requires the use of common domains to understand the aspects of a patient's appearance that the patient and clinician score differently (21).

We used a set of domains, first defined by Strasser, to explain variations in the scoring of CROM and PROM (22). Strasser set out 5 domains to objectively assess cosmetic surgical results: malposition, distortion, asymmetry, contour deformity and scarring. A strength of domain mapping is that it allows the use of distinct pre-operative and post-operative question sets to judge the cosmetic outcome.

This study aimed to determine whether there is a difference between the outcomes reported by patients and clinicians, and whether any patient-specific pre-operative considerations can predict the outcomes.

## Materials/Patients and Methods

All surgical procedures were carried out by the senior author (GLR) at the Alexandra Hospital, Mill Lane, Cheadle, Cheshire, from July 2016 to February 2020. Overall, 146 abdominoplasty procedures, as recorded on the PHIN database for hospital admission/discharges, were performed by the senior author. Patients were included if they had complete prospective data for pre-/post-operative PROM and if the Royal Free Hospital and Centre for Appearance Research (RoFCAR) psychological screening tool with pre-/post-operative standardised photographs were available that the patients consented to share (23). Thus, 146 consecutive cases were included, among which 95 were eligible for inclusion in this study, as previously described in the validation study on the MCCRO-Q (17). Fifty-one patients were not eligible for inclusion due to the unavailability of the complete data sets required for clinician completion of the CROM.

## BODY-Q

The BODY-Q questionnaire provided to the patients includes only the RCS-recommended abdomen scale (8). It consists of 7 questions and requires the patient to rate their assessment of their appearance on a scale ranging from very dissatisfied (score of one) to very satisfied (score of 4).

Each patient received one pre-operative assessment and at least one post-operative assessment. The BODY-Q was completed prospectively during every operative assessment by the patient independently without assistance or comment. The BODY-Q was provided by a non-clinical staff member and was completed by hand before consultation with the senior author to reduce patient reporting bias. Each follow-up included capturing photographs and completing the post-operative BODY-Q which occurred on average at 15 weeks, with 79% of the patients being followed-up within 12 weeks post-operation (range 6 -118 weeks). Patients were instructed to focus only on their abdomen when completing the BODY-Q, if they underwent a concomitant operation for which they were required to complete separate PROMs for the additional surgical procedure/s. The patients completed the PROMs prior to the post-operative consultation and did not review their pre-operative photos when completing the post-operative PROMs.

## MCCRO-Q

The MCCRO-Q consists of 4 distinct pre-operative questions and 4 post-operative questions, each ranked from dissatisfied, with a score of one, to a maximum score of 4 for satisfied/agree. For clinicians to assess each patient using the MCCRO-Q, photos from each pre-operative and post-operative visit were arranged in chronological order in a digital slide deck. The patients’ medical information, including the patient's age, gender, smoking status, body mass index (BMI), occupation, pre-operative and post-operative photographs, and past medical and surgical history, was reviewed for pre-operative, intraoperative and post-operative information relevant to the assessors. Clinicians were not blinded to follow-up, and the time to follow-up was included on the digital slide deck for accurate assessment of post-operative results by clinicians. Data were collected using the senior author's clinical notes and records. Assessors were supplied with the past medical and surgical history of each patient to make an informed assessment of the images. The MCCRO-Q was completed by 16 clinicians including 3 consultant plastic surgeons, 3 plastic surgical registrars, 10 junior doctors and trainees with current or previous placement at a plastic surgical hub. Inclusion in this study required the clinicians to have experience in aesthetic surgery. The effect of variation in clinician experience was assessed to determine the validity of the results. Inter-assessor reliability was assessed using intra-class coefficient with all Intra-class correlation coefficient (ICC) averages >0.5 for pre- and post-operative assessment demonstrating good agreement between assessors. The authors completed the MCCRO-Q before other clinicians to reduce bias in scoring. Every patient was assessed by at least 3 clinicians. When assessing the photographs, the clinicians were not blinded to the pre- and post-operative status and time from operation.

### Surgical methodology

All procedures were performed under general anaesthesia. All patients received prophylactic antibiotics and mechanical venous thromboembolism (VTE) prophylaxis. No patient in this series consented for pharmacological VTE prophylaxis.

All patients undergoing abdominoplasty procedures were placed in the supine position throughout. Patients undergoing the bodylift procedure were initially positioned prone and then transitioned to supine position.

Any areas requiring liposuction were infiltrated with a tumescent solution and liposuction was performed initially in most cases.

Dissection of the deep tissues was performed using electrocautery, and additional haemostasis was achieved using bipolar cautery. The Scarpa fascia layer below the umbilicus and the deep fascia posteriorly were identified and preserved in all cases.

Where abdominal plication was performed, the Scarpa fascia and deep adipose tissue were removed from the central portion below the umbilicus, and the upper abdominal flap in the epigastric area was raised in the preaponeurotic plane up to the level of the xiphoid process with a central tunnel of approximately 10 cm width. There was no undermining of upper abdominal flaps in patients undergoing fleur de lis or mini-abdominoplasty procedures.

Plication of the aponeurosis of the anterior rectus sheath was carried out in 3 layers. First, by using a 1.0 loop continuous locking Ethilon suture. This was further supplemented by a 2.0 Prolene quill and finally, by using individual 2.0 Prolene sutures. No quilting sutures, tissue fibrin glue, or drains were used in any patient. Closure was achieved in 2 layers using 2.0 Vicryl and 3.0 Monocryl. Closure generally commenced with the patient in a flexed position, but the final layer of closure was performed with the patient in a non-flexed position. If areas of fullness were found in the abdominal area, liposuction was performed using the new umbilical incision at this stage prior to suturing the umbilicus with deep 3.0 Monocryl sutures and 4.0 Vicryl rapide.

Post-operative garments were used in all cases. Patients were mobilised on the evening of the surgery, and all patients spent a night in hospital. Patients were reviewed by physiotherapy at the time of discharge, and mobilisation and exercising plans were tailored to each individual patient's recovery progress.

All patients were reviewed at 7 to 10 days post-operatively. Following this, the patients were offered follow-up at 6 weeks – 3 months, with a mean follow-up length of 15 weeks (21 weeks) and a subsequent review at 6-12 months. The senior author does not discharge patients and offers all patients an open appointment after 12 months.

In case of any concerns arose regarding a patient, they were reviewed by the senior author. If a patient reattended with a wound that required dressing or if antibiotics were given after discharge, this was recorded as a minor complication.

### Statistical analysis

The normality of continuous variables was tested using the Shapiro-Wilk test and non-parametric independent Mann–Whitney U test was used to test for significance. All non-normally distributed data were presented as median (interquartile range). All normally distributed data were presented as mean (standard deviation). Categorical variables were grouped, and percentages were calculated based on their proportion of the total for each sub-group. A p-value <0.05 was classed as significant, and p<0.002 was classed as significant when applying the Bonferroni's method of correction for multiple significance testing on a cohort. Each patient was assessed by 3 or more assessors, and average CROMs score was calculated for each patient. The BODY-Q and MCCRO-Q questions were mapped to one or more domains, which was justified independently by 2 authors (STK and GLR). Any disagreements were resolved via consensus. ICC analysis was performed on SPSS (version 29.0.0.0). All further statistical analysis was performed using the GraphPad Prism (version 9.5.0.) software.

## Results

Ninety-two patients (97%) were women and 3 patients (3%) were men. Most participants were between 38-47 years old (39%), with a mean age of 43 years (9.59) (Table 1). The mean BMI was 26.4 kg/m^2^ (3.73). Nine patients (9%) were primiparous and 69 patients (72%) were multiparous. Fifty-eight patients (61%) had previous abdominal scars due to surgery, including caesarean section. Seventy-five patients (79%) had not undergone previous plastic surgical operation. Twenty-nine patients (30%) did not undergo liposuction during their operation, 36 patients (38%) had ≤500 cc of fat tissue removed, and 30 patients (32%) had >500 cc of fat tissue removed. Rectus plication was performed in 74 patients (78%).

**Table 1:** Patient Baseline Characteristics

Gender, n (%)	
Male	3 (3)
Female	92 (97)
Age, years	
18 - 27	3 (3)
28 - 37	28 (29)
38 - 47	38 (39)
48 - 57	24 (24)
58 - 67	4 (4)
68 - 77	1 (1)
BMI, kg/m^2^ (%)	
<18.5	0
18.5 - 25	37 (39)
25 - 30	38 (40)
30 - 40	20 (21)
>40	0
Parity, n (%)	
0	14 (15)
1	9 (9)
2	43 (45)
3	21 (22)
4	5 (5)
N/A (males)	3 (3)
Previous abdominal surgery, Y/N	
Y	58 (61)
N	37 (39)
Previous plastic surgery, Y/N	
Y	20 (21)
N	75 (79)

Table 1. Demographic data on 95 patients, including gender, age (years), BMI (kg/m^2^), parity (n), previous abdominal surgery and previous plastic surgery. BMI; Body Mass Index.

The most performed abdominoplasty procedure was a full abdominoplasty in 88 patients (93%), a small vertical scar was made alongside full abdominoplasty in 6 patients (6%), 3 patients (3%) underwent Fleur De Lis, and 4 patients (4%) underwent mini-abdominoplasty. Sixty-three patients (66%) underwent a concomitant surgical operation, among which 39 underwent procedures at a separate operative site. Twelve patients (13%) were treated for minor complications such as discharge or required wound dressing or antibiotics post-discharge. There were no cases of seroma that were treated using needle aspiration, no haematomas or readmissions for infection. There were no DVT / PE cases. Three patients (3%) had a scar revision ± liposuction at a later date, following the senior author's preference to perform these procedures after a minimum of 12 months following the initial surgery.

**Figure 1:**
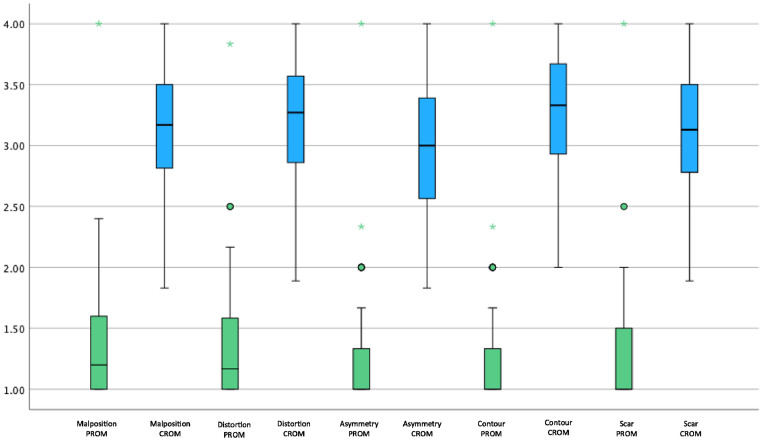
Box and Whisker plot comparing preoperative PROM and CROM domain scores. PROM; Patient-Reported Outcome Measures, CROM; Clinician-Reported Outcome Measures.

**Figure 2:**
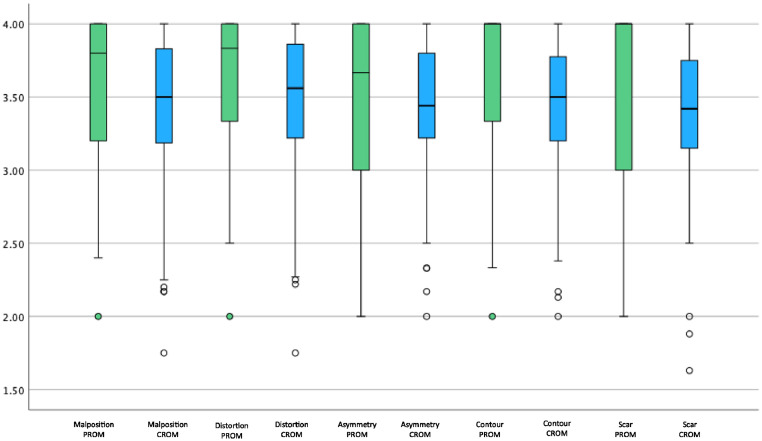
Box and Whisker plot comparing post-operative PROM and CROM domain scores. PROM; Patient-Reported Outcome Measures, CROM; Clinician-Reported Outcome Measures.

**Figure 3:**
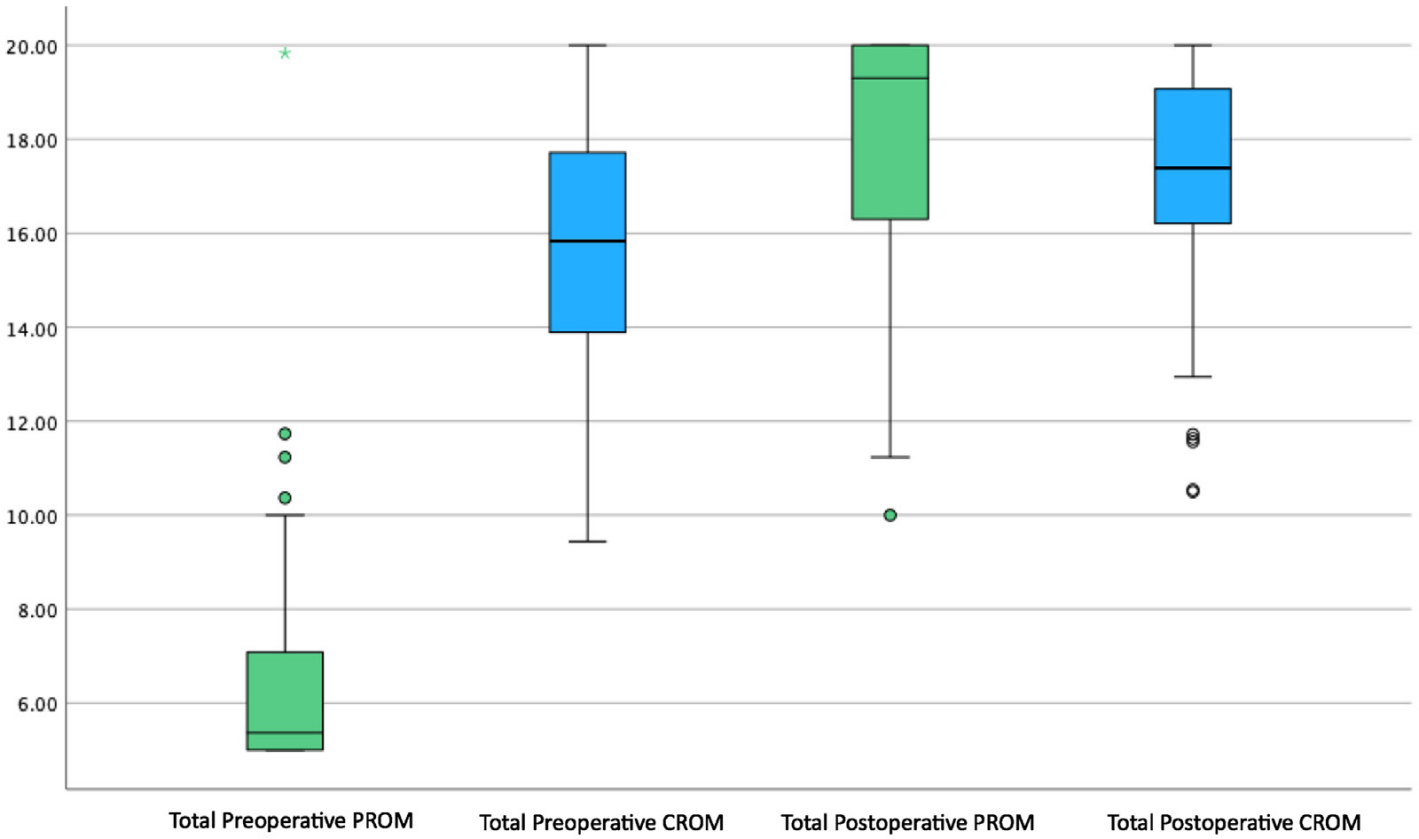
Box and Whisker plot comparing total preoperative and post-operative PROM and CROM scores. PROM; Patient-Reported Outcome Measures, CROM; Clinician-Reported Outcome Measure.

Patients rated their pre-operative appearance significantly lower across all 5 domains compared to the clinicians (p<0.001) (Table 2). Conversely, the post-operative assessment patient scores were higher compared to those of the clinicians across all domains (p<0.05). We compared overall satisfaction using a RoFCAR question that asked the patient to what extent the result matched their expectation and compared this with the same question asked to clinicians in the post-operative CROMs; we found no significant difference (p=0.427). We visualised the comparison of PROM and CROM scores preoperatively and post-operatively as a box and whisker plot to demonstrate the significantly lower pre-operative patient score and significantly increased post-operative scores across all domains (Figures 1, 2). There was a significant increase in total CROM and PROM post-operative scores relative to the pre-operative scores; however, the increase in scores by patients was significantly higher across all domains and overall (Figure 3).

**Table 2:** Comparison of 95 PROM and CROM scores by domain

	Pre-operative Assessment			Post-operative Assessment		
Domains	PROM, median (IQR)	CROM, median (IQR)	p-value	PROM, median (IQR)	CROM, median (IQR)	p-value
Malposition	1.20 (1.00 - 1.60)	3.17 (2.80 - 3.50)	<0.001⁎⁎	3.80 (3.20 - 4.00)	3.50 (3.17 - 3.83)	0.015*
Distortion	1.17 (1.00 - 1.67)	3.27 (2.83 - 3.58)	<0.001⁎⁎	3.83 (3.33 - 4.00)	3.56 (3.22 - 3.89)	0.011*
Asymmetry	1.00 (1.00 - 1.33)	3.00 (2.50 - 3.40)	<0.001⁎⁎	3.67 (3.00 - 4.00)	3.44 (3.22 - 3.80)	0.055
Contour Deformity	1.00 (1.00 - 1.33)	3.33 (2.86 - 3.67)	<0.001⁎⁎	4.0 (3.33 - 4.00)	3.50 (3.20 - 3.80)	0.011*
Scar	1.00 (1.00 - 1.50)	3.13 (2.78 - 3.50)	<0.001⁎⁎	4.00 (3.00 - 4.00)	3.42 (3.13 - 3.75)	0.022*
Total	5.37 (5.00 - 7.10)	15.83 (13.89 - 17.72)	<0.001⁎⁎	19.30 (16.23 - 20.00)	17.39 (16.17 - 19.11)	0.017*

Table 2. Comparison of 95 patient and clinician-reported outcome measures at the pre- and post-operative assessment, presented by domain and total domain score. PROM; patient-reported outcome measure. CROM; clinician-reported outcome measures. IQR; interquartile range.

⁎ p<0.05.

⁎⁎ p<0.001.

We conducted additional analyses to ensure that the follow-up time did not bias the results. By comparing the 20 patients whose follow-up post-operatively was greater than 12 weeks, we found no significant difference in clinician scoring (p=0.486) suggesting that the clinicians reliably considered follow-up time information when scoring the post-operative photograph. Inter-assessor reliability was assessed by calculating the intra-class coefficient, all ICC averages >0.5 for pre- and post-operative assessment demonstrated good agreement between the assessors, based on established agreement thresholds (24).

## Sub-analysis of the intergroup variables

We investigated the effect of obesity (BMI≥30 kg/m^2^), previous abdominal surgery, parity, age, massive weight loss methodology, total tissue removed and concomitant operation at a separate operative site on the outcomes. These factors have been shown to influence the outcome (11,25,26). The heterogeneity of the operation and patient demographics indicates that sub-analysis with small patient cohorts limits the reliability of conclusions drawn from it. The purpose of this sub-analysis was to elucidate the factors related to abdominoplasty procedure that impact PROM and CROM scores significantly.

There were 75 patients (79%) with BMI <30 kg/m^2^ and 20 patients (21%) with BMI≥30 kg/m^2^. The median BMI was 24.6 kg/m^2^ (range, 20-29 kg/m^2^) for the BMI <30 kg/m^2^ cohort and median BMI of 32 kg/m^2^ (range, 30-35 kg/m^2^) for the BMI >30 kg/m^2^ cohort. The clinicians assessed the appearance of patients with a BMI≥30 kg/m^2^ significantly lower, preoperatively and post-operatively, compared to patients with BMI <30 kg/m^2^ (p<0.002) (Table 3).

**Table 3:** Intergroup comparison of domain scores between patients with BMI ≥30 kg/m^2^, and patients with BMI <30 kg/m^2^

	Pre-operative Assessment						Post-operative Assessment					
Domains	PROM, BMI <30, median (IQR)	PROM, BMI≥30, median (IQR)	p-value	CROM, BM<30, median (IQR)	CROM, BMI≥30, median (IQR)	p-value	PROM, BMI<30, median (IQR)	PROM, BMI ≥30, median (IQR)	p-value	CROM, BM<30, median (IQR)	CROM, BMI≥30, median (IQR)	p-value
Malposition	1.20 (1.00 - 1.60)	1.00 (1.00 - 1.20)	0.020*	3.33 (3.00 - 3.67)	2.73 (2.22 - 3.00)	<0.001⁎⁎	3.80 (3.20 - 4.00)	4.00 (3.60 - 4.00)	0.095	3.65 (3.25 - 4.00)	3.21 (2.91 - 3.48)	<0.001⁎⁎
Distortion	1.17 (1.00 - 1.67)	1.00 (1.00 - 1.29)	0.037*	3.33 (3.00 - 3.67)	3.00 (2.27 - 3.15)	<0.001⁎⁎	3.83 (3.17 - 4.00)	4.00 (3.67 - 3.89)	0.080	3.67 (3.33 - 3.89)	3.28 (2.96 - 3.55)	0.003*
Asymmetry	1.00 (1.00 - 1.67)	1.00 (1.00 - 1.00)	0.092	3.15 (2.75 - 3.50)	2.50 (2.18 - 2.97)	<0.001⁎⁎	3.67 (3.00 - 4.00)	4.00 (3.54 - 4.00)	0.080	3.56 (3.33 - 3.89)	3.25 (2.90 – 3.49)	0.003*
Contour Deformity	1.00 (1.00 - 1.33)	1.00 (1.00 - 1.00)	0.086	3.40 (3.00 - 3.67)	2.93 (2.51 - 3.19)	<0.001⁎⁎	3.67 (3.00 - 4.00)	4.00 (3.67 - 4.00)	0.071	3.50 (3.25 - 3.83)	3.33 (2.84 – 3.50)	<0.001⁎⁎
Scar	1.00 (1.00 - 1.50)	1.00 (1.00 - 1.00)	0.472	3.27 (2.83 - 3.56)	2.78 (2.20 - 3.04)	<0.001⁎⁎	3.50 (3.00 - 4.00)	4.00 (3.50 - 4.00)	0.088	3.55 (3.17 - 3.83)	3.37 (2.81 – 3.63)	<0.001⁎⁎
Total	5.33 (5.00 - 7.40)	5.00 (5.00 - 5.81)	0.074*	16.53 (14.63 - 17.86)	13.89 (11.55 - 15.06)	<0.001⁎⁎	18.60 (15.53 - 20.0)	19.82 (17.95 - 20.0)	0.098	17.79 (16.50 - 19.33)	16.53 (14.48 - 17.66)	0.005*

Table 3. Intergroup comparison of PROM and CROM score by domain between 20 patients with BMI≥30 kg/m^2^ and 75 patients with BMI <30 kg/m^2^. PROM; patient-reported outcome measure. CROM; clinician-reported outcome measures. IQR; interquartile range.

⁎ p<0.05.

⁎⁎ p<0.001.

Twenty-three patients (24%) reported massive weight loss (MWL) prior to their abdominoplasty. Ten patients lost weight via diet and exercise and 13 patients lost weight via bariatric surgery. The diet and exercise group had a median reduction in BMI of 11 kg/m^2^ (range, 5.4-19.7 kg/m^2^). The bariatric surgery group had a median reduction in BMI of 9.5 kg/m^2^ (range of 4-28 kg/m^2^). There was no significant difference in the BMI of patients before MWL (p=0.196) or after MWL (p=0.493). Clinicians and patients scored the appearance of patients who had MWL via diet and exercise as having significantly better pre-operative appearance in at least one domain and these patients achieved a significantly greater clinical outcome compared to patients who underwent bariatric surgery (p<0.05) (Table 4).

**Table 4:** Intergroup comparison of PROM and CROM scores by domain between MWL patients who lost weight via diet and exercise, and bariatric surgery

	Pre-operative Assessment						Post-operative Assessment					
Domains	PROM Diet and Exercise, median (IQR)	PROM Bariatric Surgery, median (IQR)	p-value	CROM Diet and Exercise, median (IQR)	CROM Bariatric Surgery, median (IQR)	p-value	PROM Diet and Exercise, median (IQR)	PROM Bariatric Surgery, median (IQR)	p-value	CROM Diet and Exercise, median (IQR)	CROM Bariatric Surgery, median (IQR)	p-value
Malposition	1.40 (1.00 - 1.60)	1.00 (1.00 - 1.25)	0.105	3.00 (2.83 - 3.66)	2.50 (2.29 - 3.15)	0.058	3.80 (3.10 - 4.00)	3.80 (3.15 - 4.00)	0.937	3.65 (3.23 - 3.86)	3.08 (2.57 - 3.31)	0.005*
Distortion	1.33 (1.00 - 1.58)	1.00 (1.00 - 3.04)	0.173	3.17 (3.00 - 3.73)	2.77 (2.40 - 3.04)	0.015*	3.83 (3.08 - 4.00)	3.83 (3.25 - 4.00)	0.936	3.67 (3.33 - 3.91)	2.98 (2.73 - 3.49)	0.011*
Asymmetry	1.00 (1.00 - 1.67)	1.00 (1.00 - 1.00)	0.042*	3.00 (2.67 - 3.41)	2.36 (2.14 - 3.10)	0.112	3.67 (3.00 - 4.00)	3.67 (3.00 - 4.00)	0.848	3.24 (3.22 - 3.91)	3.22 (2.50 - 3.47)	0.059
Contour Deformity	1.33 (1.00 - 1.50)	1.00 (1.00 - 1.33)	0.215	3.00 (2.92 - 3.64)	2.86 (2.52 - 3.33)	0.102	4.00 (3.17 - 4.00)	3.82 (3.0 - 4.00)	0.875	3.50 (3.02 - 3.86)	3.39 (2.75 - 3.54)	0.275
Scar	1.50 (1.00 - 1.50)	1.00 (1.00 - 1.13)	0.052	3.11 (2.84 - 3.56)	2.65 (2.13 - 2.06)	0.040*	4.00 (3.00 - 4.00)	3.75 (3.00 - 4.00)	0.866	3.55 (2.92 - 3.75)	3.28 (2.75 - 3.49)	0.276
Total	6.20 (5.37 - 8.03)	5.45 (5.00 - 5.93)	0.080	15.25 (14.06 - 17.98)	14.32 (11.45 - 15.71)	0.119	19.30 (15.62 - 20.00)	18.89 (15.40 - 20.00)	0.889	17.17 (16.00 - 19.22)	15.95 (13.27 - 17.07)	0.044*

Table 4. Intergroup comparison of PROM and CROM score by domain between 10 MWL patients who lost weight via diet and exercise and 13 patients via bariatric surgery. PROM; patient-reported outcome measure. CROM; clinician-reported outcome measures. IQR; interquartile range. MWL; massive weight loss.

⁎ p<0.05 **p<0.001.

Thirty-four patients (36%) had <1000 g of fat tissue removed intraoperatively, and 61 patients (64%) had ≥1000 g of fat tissue removed intraoperatively. The median tissue removed for the <1000 g cohort was 542 g (range, 165-975 g). The median tissue removed for the >1000 g cohort was 1697.5 g (range, 1017-3600 g). Clinicians rated patients with ≥1000 g tissue removed significantly lower across all domains preoperatively (p<0.002) and rated the post-operative appearance significantly worse overall (p<0.05) (Table 5).

**Table 5:** Intergroup comparison of PROM and CROM scores by domain between patients with ≥1000 g excised fat tissue, and patients with <1000 g excised fat tissue

	Pre-operative Assessment						Post-operative Assessment					
Domains	PROM, ≥1000, median (IQR)	PROM, <1000, median (IQR)	p-value	CROM, ≥1000, median (IQR)	CROM, <1000, median (IQR)	p-value	PROM, ≥1000, median (IQR)	PROM, <1000, median (IQR)	p-value	CROM, ≥1000, median (IQR)	CROM, <1000, median (IQR)	p-value
Malposition	1.00 (1.00 - 1.25)	1.20 (1.00 - 1.80)	0.016*	2.83 (2.29 - 3.17)	3.40 (3.00 - 3.67)	<0.001⁎⁎	3.80 (3.20 - 4.00)	3.80 (3.20 - 4.00)	0.966	3.20 (2.79 - 3.69)	3.67 (3.33 - 4.00)	0.004*
Distortion	1.00 (1.00 - 1.33)	1.17 (1.00 - 1.83)	0.017*	3.00 (2.33 - 3.23)	3.47 (3.10 - 3.73)	<0.001⁎⁎	3.83 (3.33 - 4.00)	3.82 (3.33 - 4.00)	0.941	3.33 (2.94 - 3.69)	3.67 (3.33 - 3.89)	0.010*
Asymmetry	1.00 (1.00 - 1.00)	1.00 (1.00 - 1.67)	0.016*	2.67 (2.17 - 3.09)	3.25 (2.83 - 3.57)	<0.001⁎⁎	3.67 (3.00 - 4.00)	3.67 (3.00 - 4.00)	1.000	3.39 (2.89 - 3.60)	3.56 (3.33 - 3.89)	0.015*
Contour Deformity	1.00 (1.00 - 1.33)	1.00 (1.00 - 1.33)	0.049*	3.00 (2.50- 3.33)	3.50 (3.00 - 3.38)	<0.001⁎⁎	3.83 (3.33 - 4.00)	4.00 (3.25 - 4.00)	0.821	3.43 (2.97 - 3.67)	3.50 (3.29 - 3.83)	0.113
Scar	1.00 (1.00 - 1.00)	1.00 (1.00 - 1.50)	0.198	2.81 (2.33 - 3.12)	3.33 (3.00 - 3.60)	<0.001⁎⁎	3.75 (3.44 - 4.00)	4.00 (3.00 - 4.00)	0.829	3.41 (2.81 - 3.68)	3.50 (3.17 - 3.83)	0.073
Total	5.27 (5.00 - 5.89)	5.53 (5.00 - 7.63)	0.055	14.00 (11.57 - 15.87)	16.67 (15.20 - 18.06)	<0.001⁎⁎	18.89 (16.49 - 20.00)	19.30 (15.93 - 20.00)	0.844	16.62 (14.21 - 18.14)	17.75 (16.55 - 19.25)	0.017*

Table 5. Intergroup comparison of PROM and CROM score by domain between 61 patients with ≥1000 g excised fat tissue and 34 patients with <1000 g excised fat tissue. PROM; patient-reported outcome measure. CROM; clinician-reported outcome measures. IQR; interquartile range.

⁎ p<0.05

⁎⁎ p<0.001

Sixty-three patients (66%) underwent concomitant operation and 32 patients (34%) underwent only abdominoplasty. Among the 63 patients who underwent concomitant operations, 24 patients (25%) underwent additional abdominal operation, and 39 patients (41%) underwent procedures at a different operative site. Patients who had concomitant operations at a different site had a greater CROM score compared to patients who underwent abdominoplasty alone (p<0.05) (Table 6).

**Table 6:** Intergroup comparison of PROM and CROM scores by domain between patients undergoing concomitant operation at a different site and patients undergoing abdominoplasty alone

Domains	Pre-operative Assessment						Post-operative Assessment					
	PROM, Additional op, median (IQR)	PROM, No Additional op, median (IQR)	p-value	CROM, Additional op, median (IQR)	CROM, No Additional op, median (IQR)	p-value	PROM, Additional op, median (IQR)	PROM, No Additional op, median (IQR)	p-value	CROM, Additional op, median (IQR)	CROM, No Additional op, median (IQR)	p-value
Malposition	1.20 (1.00 - 1.80)	1.00 (1.00 - 1.60)	0.236	3.17 (2.67 - 3.50)	3.29 (2.83 - 3.62)	0.676	3.60 (3.20 - 4.00)	3.80 (3.25 - 4.00)	0.337	3.67 (3.25 - 4.00)	3.46 (3.19 - 3.78)	0.136
Distortion	1.33 (1.00 - 1.83)	1.00 (1.00 - 1.83)	0.231	3.22 (2.78 - 3.58)	3.30 (2.87 - 3.59)	0.829	3.67 (3.33 - 4.00)	3.83 (3.35 - 4.00)	0.322	3.67 (3.28 - 3.92)	3.43 (3.18 - 3.77)	0.061
Asymmetry	1.00 (1.00 - 1.67)	1.00 (1.00 - 1.58)	0.335	3.00 (2.50 - 3.50)	3.03 (2.54 - 3.48)	0.767	3.67 (3.00 - 4.00)	3.83 (3.04 - 4.00)	0.278	3.58 (3.38 - 3.89)	3.36 (3.13 - 3.65)	0.035*
Contour Deformity	1.00 (1.00 - 1.33)	1.00 (1.00 - 1.33)	0.629	3.33 (2.83 - 3.75)	3.29 (2.81 - 3.67)	0.676	3.67 (3.00 - 4.00)	4.00 (3.04 - 4.00)	0.248	3.63 (3.33 - 3.83)	3.33 (3.04 - 3.66)	0.017*
Scar	1.00 (1.00 - 1.50)	1.00 (1.00 - 1.0)	0.483	3.11 (2.78 - 3.50)	3.17 (2.81 - 3.52)	0.843	3.50 (3.00 - 4.00)	4.00 (3.00 - 4.00)	0.389	3.67 (3.17 - 3.88)	3.39 (3.00 - 3.70)	0.042*
Total	5.53 (5.00 - 7.47)	5.08 (5.00 - 7.17)	0.238	15.81 (13.97 - 17.77)	15.98 (13.97 - 17.77)	0.870	18.47 (15.53 - 20.00)	19.30 (15.69 - 20.00)	0.368	17.94 (16.45 - 19.56)	16.92 (15.57 - 18.40)	0.047*

Table 6. Intergroup comparison of PROM and CROM score by domain between 39 patients undergoing concomitant operation at a separate site and 32 patients undergoing abdominoplasty alone. PROM; patient-reported outcome measure. CROM; clinician-reported outcome measures. IQR; interquartile range.

⁎ p<0.05 ^⁎⁎^p<0.001

Overall, 12 patients (13%) had at least one post-operative complication and 83 patients (87%) had no complication post-operation (Table 7). There was no significant difference in the BMI of patients who had a complication (p=0.461). Patients with post-operative complications were considered to have worse clinical outcomes (p=0.036); however, patients found no significant difference in the outcome.

**Table 7:** Intergroup comparison of PROM and CROM scores by domain between patients who had at least one post-operative complication and patients without complication post-operatively

Domains	Pre-operative Assessment						Post-operative Assessment 1					
	PROM Complication, median (IQR)	PROM No Complication, median (IQR)	p-value	CROM Complication, median (IQR)	CROM No Complication, median (IQR)	p-value	PROM Complication, median (IQR)	PROM No Complication, median (IQR)	p-value	CROM Complication, median (IQR)	CROM No Complication, median (IQR)	p-value
Malposition	1.00 (1.00 - 1.60)	1.00 (1.00 - 2.00)	0.922	2.96 (2.71 - 3.57)	3.25 (2.80 - 3.50)	0.360	3.90 (3.35 - 4.00)	3.80 (3.20 - 4.00)	0.242	3.40 (2.54 - 3.67)	3.50 (3.20 - 3.88)	0.119
Distortion	1.98 (1.00 - 1.96)	1.67 (1.00 - 1.67)	0.941	3.03 (2.79 - 3.63)	3.27 (2.89 - 3.58)	0.403	3.83 (3.33 - 4.00)	3.83 (3.33 - 4.00)	0.380	3.36 (2.59 - 3.67)	3.56 (3.25 - 3.89)	0.057
Asymmetry	1.00 (1.00 - 1.83)	1.00 (1.00 - 1.33)	0.705	2.83 (2.37 - 2.67)	3.08 (2.67 - 3.50)	0.195	3.83 (3.17 - 4.00)	3.67 (3.00 - 4.00)	0.549	3.32 (2.64 - 3.48)	3.56 (3.25 - 3.89)	0.023*
Contour Deformity	1.00 (1.00 - 1.25)	1.00 (1.00 - 1.33)	0.545	3.00 (2.76 - 3.50)	3.33 (3.00 - 3.67)	0.237	4.00 (3.17 - 4.00)	3.67 (3.33 - 4.00)	0.562	3.32 (2.64 - 3.48)	3.50 (3.25 - 3.83)	0.035*
Scar	1.00 (1.00 - 1.00)	1.00 (1.00 - 1.50)	0.617	3.00 (2.32 - 3.44)	3.17 (2.78 - 3.50)	0.284	4.00 (3.13 - 4.00)	4.00 (3.00 - 4.00)	0.650	3.17 (2.69 - 3.40)	3.17 (2.69 - 3.40)	0.043*
Total	5.08 (5.00 - 8.04)	5.37 (5.00 - 7.10)	0.746	14.65 (13.04 - 17.41)	16.04 (13.89 - 17.78)	0.260	19.30 (16.27 - 20.00)	19.17 (16.23 - 20.00)	0.610	16.56 (13.23 - 17.62)	17.46 (16.28 - 19.17)	0.036*

Table 7. Intergroup comparison of PROM and CROM score domain between 12 patients who had at least one post-operative complication and 83 patients without complication post-operatively. PROM; patient-reported outcome measure. CROM; clinician-reported outcome measures. IQR; interquartile range.

⁎ p<0.05 ^⁎⁎^p<0.001

Patients who underwent previous abdominal surgery and patients with previous pregnancies showed no significant difference in patient or clinician total domain scores based on their appearance preoperatively and post-operatively. Thirty-seven patients (39%) had no previous abdominal surgery and 58 patients (61%) had previous abdominal surgery; thus, scarring was present preoperatively (Table 8). Fourteen patients (15%) were nulliparous and 78 (85%) were primiparous or multiparous; 3 male patients (3%) were excluded from this sub-analysis (Table 9). Clinicians and patients found no overall significant difference in appearance when grouped by age**;** 23 patients (24%) were aged ≥50 years and 72 patients (76%) were aged <50 years old (Table 10).

**Table 8:** Intergroup comparison of domain scores between patients with previous abdominal surgery and patients with no history of abdominal surgery

	Pre-operative Assessment						Post-operative Assessment					
Domains	PROM, Previous abdominal surgery, median (IQR)	PROM, No previous abdominal surgery, median (IQR)	p-value	CROM, Previous abdominal surgery, median (IQR)	CROM, No previous abdominal surgery, median (IQR)	p-value	PROM, Previous abdominal surgery, median (IQR)	PROM, No previous abdominal surgery, median (IQR)	p-value	CROM, Previous abdominal surgery, median (IQR)	CROM, No previous abdominal surgery, median (IQR)	p-value
Malposition	1.10 (1.00 - 1.40)	1.20 (1.00 - 1.75)	0.271	3.17 (1.00 - 1.40)	3.38 (2.83 - 3.67)	0.097	3.80 (3.20 - 4.00)	3.80 (3.20 - 4.00)	0.879	3.50 (3.17 - 3.83)	3.65 (3.23 - 3.88)	0.432
Distortion	1.17 (1.00 - 1.50)	1.17 (1.00 - 1.83)	0.443	3.24 (2.78 - 3.59)	3.33 (3.0 - 3.73)	0.049*	3.83 (3.33 - 4.00)	3.83 (3.33 - 4.00)	0.879	3.47 (3.22 -3.81)	3.67 (3.20 - 3.92)	0.401
Asymmetry	1.00 (1.00 - 1.33)	1.00 (1.00 - 1.67)	0.301	3.00 (2.50 - 3.67)	3.10 (2.73 - 3.67)	0.145	3.67 (3.00 - 4.00)	3.67 (3.08 - 4.00)	0.941	3.44 (3.22 - 3.79)	3.50 (3.20 - 3.90)	0.509
Contour Deformity	1.00 (1.00 - 1.33)	1.00 (1.00 - 1.33)	0.116	3.62 (3.33 - 3.62)	3.40 (2.93 - 3.65)	0.165	3.83 (3.25 - 4.00)	4.00 (3.33 - 4.00)	0.702	3.50 (3.17 - 3.76)	3.43 (3.20 - 3.83)	0.745
Scar	1.00 (1.00 - 1.00)	1.00 (1.00 - 1.50)	0.178	3.11 (2.67 - 3.44)	3.25 (2.91 - 3.67)	0.072	4.00 (3.00 - 4.00)	4.00 (3.25 - 4.00)	0.599	3.46 (3.17 - 3.81)	3.40 (3.09 - 3.75)	0.968
Total	5.37 (5.00 - 6.41)	5.83 (5.00 - 7.50)	0.301	17.30 (16.16 - 19.01)	16.50 (14.57 - 18.34)	0.218	19.30 (15.66 - 20.00)	19.17 (16.38 - 20.00)	0.967	17.30 (16.16 - 19.01)	17.70 (16.12 - 19.33)	0.609

Table 8. Intergroup comparison of PROM and CROM score by domain between 58 patients with previous abdominal surgery and 37 patients with no history of abdominal surgery. PROM; patient-reported outcome measure. CROM; clinician-reported outcome measures. IQR; interquartile range.

⁎ p<0.05 **p<0.001

**Table 9:** Intergroup comparison of PROM and CROM scores by domain between nulliparous patients and patients with ≥1 pregnancy

	Pre-operative Assessment						Post-operative Assessment					
Domains	PROM, Nulliparous, median (IQR)	PROM, ≥1 Pregnancy, median (IQR)	p-value	CROM, Nulliparous, median (IQR)	CROM, ≥1 Pregnancy, median (IQR)	p-value	PROM, Nulliparous, median (IQR)	PROM, ≥1 Pregnancy, median (IQR)	p-value	CROM, Nulliparous, median (IQR)	CROM, ≥1 Pregnancy, median (IQR)	p-value
Malposition	1.30 (1.00 - 1.70)	1.20 (1.00 - 1.45)	0.306	2.96 (2.50 - 3.38)	3.25 (2.82 - 3.53)	0.081	3.80 (3.35 - 4.00)	3.80 (3.20 - 4.00)	0.976	3.40 (2.79 - 3.68)	3.25 (2.82 - 3.53)	0.523
Distortion	1.25 (1.00 - 1.75)	1.67 (1.00 - 1.54)	0.411	3.03 (1.70 - 1.54)	3.27 (2.82 - 3.59)	0.156	3.83 (3.29 - 4.00)	3.83 (3.33 - 4.00)	0.923	3.41 (2.94 - 3.67)	3.56 (3.22 - 3.89)	0.090
Asymmetry	1.00 (1.00 - 1.50)	1.00 (1.00 - 1.33)	0.600	2.92 (2.42 - 3.03)	3.08 (2.60 - 3.50)	0.052	3.67 (3.33 - 4.00)	3.67 (3.00 - 4.00)	0.987	3.36 (3.06 - 3.57)	3.47 (3.22 - 3.85)	0.078
Contour Deformity	1.33 (1.00 - 1.50)	1.00 (1.00 - 1.33)	0.960	3.00 (2.81 - 3.50)	3.33 (2.85 - 3.67)	0.191	3.83 (3.33 - 4.00)	4.00 (3.29 - 4.00)	0.916	3.32 (2.83 - 3.63)	3.50 (3.19 - 3.83)	0.117
Scar	1.00 (1.00 - 1.63)	1.00 (1.00 - 1.00)	0.109	3.00 (1.00 - 1.63)	3.20 (2.78 - 3.51)	0.100	3.75 (3.50 - 4.00)	4.00 (3.00 - 4.00)	0.084	3.32 (2.81 - 3.72)	4.00 (3.00 - 4.00)	0.210
Total	5.95 (5.28 - 7.70)	5.37 (5.00 - 7.08)	0.127	15.21 (13.33 - 16.16)	16.09 (13.88 - 17.78)	0.098	18.89 (16.81 - 20.00)	19.30 (16.04 - 20.00)	0.989	16.67 (15.07 - 18.04)	17.39 (16.23 - 19.17)	0.110

Table 9. Intergroup comparison of PROM and CROM score by domain between 14 nulliparous patients and 78 patients with ≥1 pregnancy. PROM; patient-reported outcome measure. CROM; clinician-reported outcome measures. IQR; interquartile range.

*p<0.05

**p<0.001

**Table 10:** Intergroup comparison of PROM and CROM scores by domain between patients aged ≥50 years and patients aged <50 years

	Pre-operative Assessment						Post-operative Assessment					
Domains	PROM, AGE ≥50, median (IQR)	PROM, AGE <50, median (IQR)	p-value	CROM, AGE ≥50, median (IQR)	CROM, AGE <50, median (IQR)	p-value	PROM, AGE ≥50, median (IQR)	PROM, AGE <50, median (IQR)	p-value	CROM, AGE ≥50, median (IQR)	CROM, AGE <50, median (IQR)	p-value
Malposition	1.20 (1.00 - 1.70)	1.10 (1.00 - 1.55)	0.331	3.00 (2.38 - 3.42)	3.25 (2.83 - 3.62)	0.165	4.00 (3.40 - 4.00)	3.80 (3.20 - 4.00)	0.233	3.0 (2.38 - 3.42)	3.56 (3.22 - 3.89)	<0.001*
Distortion	1.17 (1.00 - 1.83)	1.70 (1.00 - 1.50)	0.391	3.17 (2.31 - 3.50)	3.33 (2.90 - 3.65)	0.246	4.00 (3.42 - 4.00)	3.83 (3.25 - 4.00)	0.256	3.17 (2.31 - 3.50)	3.33 (2.90 - 3.65)	0.246
Asymmetry	1.00 (1.00 - 1.67)	1.00 (1.00 - 1.33)	0.385	3.00 (2.26 - 3.29)	3.12 (2.67 - 3.50)	0.108	4.00 (3.33 - 4.00)	3.67 (3.00 - 4.00)	0.185	3.00 (2.25 - 4.00)	3.12 (2.67 - 3.50)	0.108
Contour Deformity	1.00 (1.00 - 1.67)	1.00 (1.00 - 1.33)	0.181	3.17 (2.54 - 3.50)	3.33 (3.00 - 3.67)	0.168	4.00 (3.33 - 4.00)	3.67 (3.33 - 4.00)	0.434	3.17 (2.54 - 3.50)	3.33 (3.0 - 3.67)	0.168
Scar	1.00 (1.00 - 1.50)	1.00 (1.00 - 1.00)	0.045*	3.08 (2.58 - 3.42)	3.20 (2.78 - 3.52)	0.171	4.00 (3.50 - 4.00)	3.50 (3.00 - 4.00)	0.306	3.08 (2.58 - 3.43)	3.20 (2.78 - 3.52)	0.171
Total	5.83 (5.00 - 8.47)	5.37 (5.00 - 6.93)	0.230	15.25 (11.58 - 16.80)	16.11 (13.97 - 17.77)	0.169	20.00 (17.23 - 20.00)	19.05 (15.83 - 20.00)	0.269	16.63 (15.50 - 19.11)	17.58 (16.46 - 19.02)	0.320

Table 10. Intergroup comparison of PROM and CROM score by domain between 23 patients aged ≥50 years and 72 patients aged <50 years. PROM; patient-reported outcome measure. CROM; clinician-reported outcome measures. IQR; interquartile range.

⁎ p<0.05 **p<0.001

## Discussion

Currently, PROMs are not routinely compared to CROMs. Interestingly, patients perceive greater improvement in outcomes compared to clinicians when comparing PROM and CROM results. Patients rate their pre-operative appearance to be significantly worse than clinicians (p<0.002) and their post-operative outcomes to be significantly better than clinicians (p<0.05) (Table 2). The overall outcome improvement reported by patients was not significantly affected by any measure we tested in the sub-analysis, but clinician scores were affected by BMI, MWL method, amount of excess tissue removed, concomitant operations, and post-operative complications.

Patients with a pre-operative BMI >30 kg/m^2^ or those who had >1000 g of excess fat tissue removed had significantly lower post-operative CROM scores (Table 3; Table 5), whereas patients did not perceive significant difference. Similarly, patients who achieved MWL via diet and exercise versus bariatric surgery had significantly better clinical outcomes preoperatively and post-operatively (Table 4); however, the patient-reported outcomes were not affected by the weight loss method. This suggests that patients may achieve better clinically assessed outcome if they reduce their pre-operative weight or lose weight via diet and exercise. Moreover, in these patient groups, the expectations from the surgery are less, and the patients accept outcomes that the clinicians would not be satisfied with.

Patients who had concomitant operations at a different site received better clinical outcome scores (p<0.05) with improved symmetry, contours and scaring, whereas patients described no difference in outcomes (Table 6). No difference was found in the complication rates between patients with multiple procedures or abdominoplasty alone. It is believed that patients may have a worse outcome or increased complications if they undergo multiple procedures; however, this has not been proven in this analysis. In this study, patients were only offered multiple procedures when the perceived operative time was <4 hours, and patients undergoing multiple procedures involved more than one surgeon with concomitant procedures performed simultaneously. It is generally accepted that increased operative time can lead to increased complications; therefore, it is important to consider this when offering multiple surgeries in one sitting (27).

Patients with complications had significantly lower CROM scores than patients who described no complications (Table 7). It is imperative that if a complication arises, patients is seen promptly, reassured and treated without delay. In this study, any patient who described a complication was seen within 24 hours by the senior author and offered further post-operative visits until the patient chose not to reattend. In some post-operative scenarios, it became evident that additional surgery may be required to improve an outcome. Acknowledging this at an early stage can help with the patient's subsequent management. Often, clinicians feel or worry that a patient who developed a complication may be dissatisfied. The senior author believes that patients with complications will only become dissatisfied with an outcome if the complication is not acknowledged and treated appropriately. Moreover, the senior author considers that patients who develop complications and are treated appropriately may rate their outcomes better than those who did not develop any complications. There were no cases in this study where the patient was dissatisfied when the clinician was satisfied with the result.

Furthermore, clinicians should be aware of their own clinical expectations, which can affect how they view the results and if they offer additional/revisionary surgery. It is the senior author's belief that only the patient should determine whether they wish to undergo any additional/revisional surgery to improve an outcome; therefore, he does not offer additional/revisional surgery to improve a clinical outcome solely. The rates of revisions in this study were only 3%. The senior author acknowledges the cases of several patients whose outcomes could have been improved via additional revisional surgery. If the patients had achieved the result they wished for, additional/revisional surgery was not offered. The rates of revision may also be lower than those reported elsewhere as patients are additionally charged for scar revisions (although the senior author rarely charged surgical or anaesthetic fee). Thus, there may have been patients who wished to improve their outcome but did not wish to pay an additional fee.

In conclusion, we have demonstrated that clinicians underestimate the improvements in outcomes that patients find satisfactory and need to be aware of their own selection bias when consulting with patients preoperatively, as patients found improvement regardless of the pre-operative or post-operative variable tested.

## Conflict of Interest

None. No authors had a conflict of interest.
